# Estimating variations in the use of antibiotics in primary care: Insights from the Tuscany region, Italy

**DOI:** 10.1002/hpm.3388

**Published:** 2021-11-20

**Authors:** Claire Willmington, Milena Vainieri, Chiara Seghieri

**Affiliations:** ^1^ Laboratorio Management e Sanità Institute of Management Scuola Superiore Sant’Anna Pisa Italy

**Keywords:** antibiotic use, antibiotics, performance evaluation system, practice variation

## Abstract

**Background:**

Practice variation is a well‐known phenomenon that affects all aspects of healthcare delivery and leads to suboptimal health outcomes as well as poor resource allocation. Given the global rise of antimicrobial resistance, practice variation is of particular concern when it comes to the prescription of antibiotics. A growing number of healthcare systems are tackling this issue at all levels of healthcare governance.

**Aims and objectives:**

This study sought to estimate the variation in antibiotic use across different levels of Tuscany's primary care, and assess the extent to which the organization of primary care delivery is responsible for this variation.

**Methods:**

We analysed the performance and variation for seven indicators related to the use of antibiotics at three levels of healthcare governance: (i) the clinician level (2619 general practitioners [GPs]); (ii) the peer‐group level (all 116 GP group practices) and (iii) the institutional level (all 26 health districts). For the statistical analysis, we built three‐level mixed effects models that were fitted with 2619 GPs, 116 GP group practices and 26 health districts.

**Results:**

The multi‐level models suggested that the grand majority of the variation in antibiotic use was located at the GP level (75% to 97%). However, the percentage of variation associated with GP group practices and health districts ranged from 3% to 25%, depending on the type of indicator analysed.

**Conclusion:**

While the variation was found to be in large part due to differences between GPs themselves, the influence exerted by peer groups and institutional mechanisms does have a significant impact as well. Further research needs to be conducted regarding the institutional and contextual factors that prompt GPs to harmonize their prescribing behaviour in line with best practices and lead to not only improved patient outcomes but also large cost‐savings.

## Highlights

- Unwarranted practice variation in antibiotic prescribing is a contributing factor to the spread of antimicrobial resistance worldwide.
- The variation on antibiotics' consumption in Tuscany was found to be in large part due to differences between general practitioners [GPs].
- The influence exerted by GP group practices and districts has a significant impact on the variation as well.
- Future studies should investigate both institutional and contextual factors that lead GPs to harmonize their prescribing behaviour in line with best practices.

## 1 INTRODUCTION

It is fair to say, that few technologies have changed the world of medicine as antibiotics have since their discovery in the 20th century. While they have proven lifesaving when treating bacterial infection, their imprudent use has led to the global rise of antimicrobial resistance (AMR). Overuse, underuse and inadequate use of antibiotics have all contributed to this phenomenon.^1^ Recent studies have estimated that the large majority of antibiotics are prescribed in primary care.^2^ Furthermore, 23% of antibiotic prescriptions were found to be inappropriate.^3^ Although efforts were made to establish guidelines promoting best practices, practitioners' behaviour does not necessarily reflect these guidelines when prescribing antibiotics. In the case of Italy, the OECD reported it as being one of countries with the highest number of antibiotics prescribed in primary care.^4^

International studies have shown that within‐country variations in antibiotic prescribing could not be explained by differences in prevalence of underlying health conditions alone.^5^, ^6^, ^7^, ^8^ Similarly, Italian studies investigating inter‐ and intra‐regional variations in antibiotic prescribing have also suggested that variation could not be explained by differing patients' needs only.^9^, ^10^, ^11^, ^12^, ^13^

It is widely accepted that varying treatment patterns for similar health conditions can have many different sources. Practice variation across regions, hospitals or physicians is a well‐known phenomenon that has been studied and documented since the 1970s. Historically, practice variation was attributed to the practitioner's preferences or habits when deciding on how to proceed with a patient's treatment in the face of medical uncertainty.^14^ Later research, however, suggested that high practice variation occurred even if medical uncertainty was low.^15^, ^16^ This led to the ‘opportunities and constraints’ hypothesis which emphasizes on the importance of differences in the social and organizational context of the practice.^17^ These differences include the practice organization (group vs. solo practice), type of payment (fee‐for service, capitation) and availability of medical supply in the area.^15^ Furthermore, the patients' and clinicians' respective agencies may also play a role in practice variation, as clinical decisions may be based on patient expectations or characteristics such as sex, age and socio‐economic status.^18^ Practice variation is not necessarily detrimental if it aligns with available evidence and patient needs including informed choices. In fact, given the rise of personalized care, one could expect an increase in practice variation over the upcoming years. On the other hand, unwarranted practice variation cannot be reflective of patient needs and signals a deviation from evidence base. Studies have suggested that the following clinician‐related factors were associated with prescription of antibiotics in primary care: the clinician's workload (number of consultations and patients), age, trainer status, propensity to prescribe medication generally and contact with pharmaceutical representatives.^6^, ^19^, ^20^, ^21^ Patient characteristics as well as prior treatment patterns—including age; sex; type of symptoms/diagnosis; preferences and prior antibiotic treatment—have also shown to influence antibiotic prescribing.^6^, ^21^, ^22^

This study makes use of performance data, aggregated at the clinician level, to estimate the variation in antibiotic use across different levels of Tuscany's primary care; assess the extent to which the organization of primary care delivery is responsible for this variation and identify potential factors that could have an impact on the relationship between primary care organization and variation in antibiotic use.

### 1.1 Governance mechanisms to manage performance in antibiotics use in Italy

As for many countries, improving healthcare quality at reduced costs has been high on Italy's agenda. The Italian healthcare system follows the Beveridge model, whereby health services are delivered free of charge. Given the decentralized nature of the Italian system, each of the 21 Italian regions retains a certain degree of autonomy in terms of organizing, budgeting and planning for health care.

In recent years, the national health legislation has focused on primary care organization and endorsed the creation of a number of primary care group practices such as the *Aggregazioni Funzionali Territoriali* (AFTs). Group practices have been adopted by several countries as a way to tackle the rise of complex chronic conditions and put greater accountability on general practitioners (GPs). The theory goes, GPs working in the same group will be more likely to exchange information and influence one another regarding treatment decisions and clinical practice.^15^ In Italy, GPs are self‐employed, funded on a capitation basis and in large part responsible for the prescription of drugs including antibiotics. Those working in the same AFT are expected to apply the principles of clinical governance, which includes continually improving the quality of services and maintaining high standards of care. Eliminating unwarranted practice variation is one of the AFTs' main goals.^23^, ^24^

### 1.2 Study setting

The Tuscany region is in the centre of Italy and has a population of 3.7 million. There are 2619 GPs grouped into 116 AFTs, each of them consisting of 20–25 GPs serving a population of around 28,000. The AFTs' activity is coordinated along with the services provided by the 26 health districts dispersed across the three main local health authorities. To monitor and assess the performance of the regional healthcare system, the region adopted since 2004 a multidimensional performance evaluation system (PES).

More specifically, the regional PES measures multiple domains and dimensions of performance in primary care at the district and AFT level including, among others, the use of medicines.^25^ The indicators chosen to assess performance adopt a managerial perspective that appeals to both managers and policy makers, thus encouraging organizational improvement.^26^ The data related to performance of districts and AFTs is publically available via both an online platform and reports that are regularly updated. With the use of effective data visualization, different stakeholders, ranging from patients to policy‐makers, are able to identify strengths and weaknesses of their local healthcare system, and subsequently take appropriate action.^27^ Furthermore, stakeholders from different levels of healthcare governance are often brought together to discuss results from the PES, thus providing greater stimulation for performance improvement.^26^

## 2 METHODS

### 2.1 Data source

Our analysis was based on different sources of administrative health data of the Tuscany region for the year 2018. These different sources include:

- Demographic data related to the population over 16 being served by the AFT system.
- Data related to the characteristics of the GPs practising in the Tuscany region.
- Performance data aggregated at the GP level for indicators related to the use of antibiotics in patients over 16.

The patients' anonymous IDs enabled interlinkages between the above databases without disclosing the patient's identity nor any other sensitive information. The presence of such safety measures waived the need for ethical approval.

### 2.2 Variables

#### 2.2.1 Dependent variables

In terms of dependent variables, we selected seven performance indicators directly related to the use of antibiotics in adult patients over 16. These indicators are described below in Table 1. We used performance data at the individual GP level, amounting to 2619 GPs, as well as at the AFT level, covering all 116 AFTs across the Tuscany region.

**TABLE 1:** Performance indicators related to the use of antibiotics

Indicator code	Indictor description
Indicator 1	Total consumption of antibiotics expressed in DDD per 1000 inhabitants per day
Indicator 1.a	Consumption of fluoroquinolones expressed in DDD per 1000 inhabitants per day
	Fluoroquinolones cause serious side effects in different organs including tendons, muscles, joints and the nervous system. The European Medicines Agency (EMA) recommends restricting their use.^28^, ^29^
Indicator 2	Incidence of injectable antibiotics
Indicator 3	Percentage of amoxicillin based antibiotics among all amoxicillin and amoxicillin plus clavulanic acid combination based antibiotics
	Combinations of amoxicillin with clavulanic acid are known to irreversibly cause liver damage in elderly patients when administered for a prolonged period.^30^
Indicator 4	Percentage of fluoroquinolones among all antibiotics
Indicator 5	Percentage of macrolides among all antibiotics
	Macrolides have been found to be associated with increased resistance.^31^
Indicator 6	Percentage of cephalosporins (third‐generation antibiotics) among all antibiotics
	Resistance to cephalosporin is a growing concern as studies have found the presence of cephalosporin‐resistant organisms in patients from different countries.^32^, ^33^

#### 2.2.2 Explanatory variables

The explanatory variables at the individual GP level were the following: age and sex of GPs, as well as the number and average age of patients treated per GP. At the AFT level, the explanatory variables related to demographic characteristics of the area (average age of GPs, average age of patients and percentage of patients over 65), as well as the size of the AFTs (number of GPs) and number of patients registered per AFT.

### 2.3 Statistical analysis

To identify the presence of variation in the use of antibiotics and assess more fully the relationship between the organization of primary care delivery and antibiotic use, we built three‐level mixed effect models. We first tested the normality of data distribution using descriptive analysis and transformed the data into a log form when needed. Three‐level mixed effect models were fitted with GPs (*n* = 2619) at level 1, AFTs (*n* = 116) at level 2 and health districts (*n* = 26) at level 3. Initially, we ran a random intercept model empty (model 1) and estimated the variances and intraclass correlation coefficients (ICCs) at each level. We then included the level‐1 explanatory variables (model 2) and the level‐2 explanatory variables (model 3). We repeated the procedure for all seven outcome variables. Analyses were performed using STATA Data Analysis and Statistical Software.

## 3 RESULTS

**FIGURE 1:**
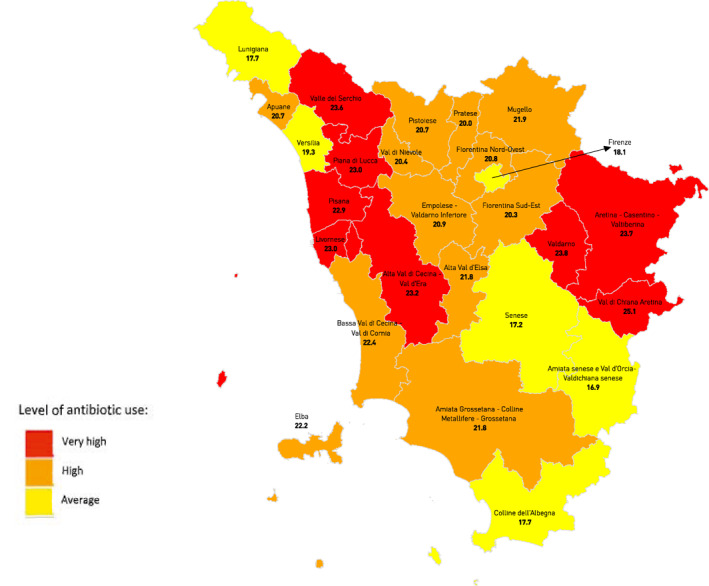
Map of Tuscany’s total consumption of antibiotics (in DDD/1000 inhabitant/day) across its 26 health districts in 2018

This study's sample included 2619 GPs working across Tuscany's 116 AFT. As shown in Table 2, there was on average 24 GPs per AFT with an average age of 61 years. On average, the consumption of antibiotics was of 21 DDD per inhabitants‐days (range 15.82–26.9) across Tuscany's AFTs. Interestingly, the average consumption of fluoroquinolones was of 3 DDD per inhabitants‐days (range 1.74–5.06), representing around 15% of total antibiotic use (Table 2). The geographical variation in the consumption of all antibiotics in Tuscany is displayed on Figure 1.

**TABLE 2:** Descriptive statistics

Variable	Mean	StD	Min	Max
District level (*N* = 26)				
Number of AFTs per district	4.46	2.27	1	10
AFT level (*N* = 115)				
Number of GPs per AFT	24	6	7	37
Number of patients treated per AFT	27,856	6955	8580	42,564
Dependent variables				
Total consumption of antibiotics (in DDD per 1000 inhabitants per day)	21	2.74	15.82	26.91
Consumption of fluoroquinolones (in DDD per 1000 inhabitants per day)	3.01	0.62	1.75	5.06
Incidence of injectable antibiotics	2.38%	0.60%	1.15%	3.8%
Percentage of amoxicillin based antibiotics among all amoxicillin and amoxicillin plus clavulanic acid combination based antibiotics	14.40%	6.43%	3.29%	34.06%
Percentage of fluoroquinolones among all antibiotics	14.74%	2.42%	10.65%	21.06%
Percentage of macrolides among all antibiotics	17.95%	2.24%	12.64%	23.10%
Percentage of cephalosporins (third‐generation antibiotics) among all antibiotics	7.56%	1.92%	3.70%	13.51%
GP level (*N* = 2619: Female = 815 (31%); Male = 1804 (69%))				
Average age of GPs	61.14	7.26	32	73
Average age of patients per GP	52.79	3.38	36.62	64.95
Average number of patients treated per GP	1152	404	1	1886
Dependent variables				
Total consumption of antibiotics (in DDD per 1000 inhabitants per day)	22.77	22.37	0.30	306.40
Consumption of fluoroquinolones (in DDD per 1000 inhabitants per day)	3.12	3.08	0.02	42.28
Incidence of injectable antibiotics	2.3%	1.28	0.14%	11.44%
Percentage of amoxicillin based antibiotics among all amoxicillin and amoxicillin plus clavulanic acid combination based antibiotics	14.89%	11.72	0.31%	76.12%
Percentage of fluoroquinolones among all antibiotics	14.30%	4.94	2.95%	47.27%
Percentage of macrolides among all antibiotics	18.14%	6.14	3.82%	50.91%
Percentage of cephalosporins (third‐generation antibiotics) among all antibiotics	7.60%	4.06	0.99%	33.90%

Abbreviations: AFT, *Aggregazioni Funzionali Territoriali*; GP, general practitioner,

The ICC estimates resulting from our multi‐level analysis are displayed in Table 3. In the empty models, the percentage of variation associated with AFTs and health districts ranged from 3% to 25%. In other words, the models suggest that 75%–97% of the observed variation was due to differences between GPs. It is worth noting that the ICC estimates for the total consumption of antibiotics and fluoroquinolones are considerably smaller than for other indicators, namely the percentage of amoxicillin based antibiotics and cephalosporins (Table 3). This could be partly explained by the fact that the values for the first two indicators (total consumption of antibiotics and fluoroquinolones) are absolute and were inserted in their log form into model using a maximum‐likelihood estimation method. Whereas, the values for the other five indicators are percentages that were kept untransformed when inserted into the model. Notably, the AFT's effect on total variation is almost non‐existent for the total consumption of antibiotics and fluoroquinolones (see Appendix 3 in supporting information S2), thus resulting in ICC1 and ICC2 being identical in both cases. Although adjusting the model for explanatory variables did not significantly affect the ICCs (Table 3), both GP and AFT level explanatory variables induced a significant decrease in the total variation for several indicators: 17% for total consumption of antibiotics and 7% for the percentage of fluoroquinolones and injectable antibiotics (Appendix 1 in supporting information S2). Additionally, our models suggested that the age of GPs was associated with the consumption of all antibiotics and fluoroquinolones as well as the percentage of injectable antibiotic and amoxicillin (Appendix 2 in supporting information S2). Patient age, on the other hand, was also significantly associated with performance on several indicators, namely the consumption of fluoroquinolones and percentage of injectable antibiotics and third‐generation cephalosporins. As for the number of patients registered per GP, it was negatively associated with the consumption of fluoroquinolones and the percentage of injectable antibiotics and third‐generation cephalosporins (Appendix 2 in supporting information S2).

**TABLE 3:** Multilevel model: random effects—the ICCs (intraclass correlation coefficients) estimates

	Model 1 (empty model)	Model 2 (with level‐1 explanatory variables)	Model 3 (with level‐1 plus level‐2 explanatory variables)
Total consumption of antibiotics expressed in DDD per 1000 inhabitants per day			
ICC1	0.0264330	0.0297268	0.0303375
ICC2	0.0264343	0.0368562	0.0314836
Consumption of fluoroquinolones expressed in DDD per 1000 inhabitants per day			
ICC1	0.0354159	0.0432216	0.0473953
ICC2	0.0354159	0.0483858	0.0478619
Incidence of injectable antibiotics			
ICC1	0.1286437	0.1322736	0.1252857
ICC2	0.1576376	0.1645001	0.1548005
Percentage of amoxicillin‐based antibiotics among all amoxicillin and amoxicillin acid plus clavulanic combination based antibiotics			
ICC1	0.1508553	0.1507281	0.1380333
ICC2	0.2498442	0.2495006	0.2358899
Percentage of fluoroquinolones among all antibiotics			
ICC1	0.1312849	0.1185935	0.1144413
ICC2	0.1529252	0.13882	0.1317041
Percentage of macrolides among all antibiotics			
ICC1	0.0638638	0.0647692	0.0622947
ICC2	0.0930864	0.0944971	0.0911328
Percentage of cephalosporins (third‐generation antibiotics) among all antibiotics			
ICC1	0.1336737	0.1383483	0.1330545
ICC2	0.1668388	0.1750876	0.1671652

*Note*: ICC1 refers to the ICC between GPs practicing in the same health district. ICC2 refers to the ICC between GPs practicing in the same AFT (and therefore the same health district).

## 4 DISCUSSION

This study contributes to an existing line of research investigating variation in antibiotic use within a geographical area.^34^ Our results reported an average antibiotic consumption of 21 DDD per inhabitants‐days in the Tuscany region. It is worth noting that this figure accounts for the use of antibiotics that were either prescribed by a GP or directly purchased over the counter by the user. The relatively high consumption of broad‐spectrum fluoroquinolones and third‐generation cephalosporins reported in this study echoes previous research that had found Italy to have one of the highest consumption of these molecules in Europe.^35^ Given the higher use of broad‐spectrum amoxicillin plus clavulanic acid combination as opposed to amoxicillin alone is concerning, especially because the combinations are associated with the spread of AMR. Furthermore, the use of amoxicillin among amoxicillin and amoxicillin/clavulanic acid combinations varies widely across the region (3.29%–34%).

The results from the multi‐level models reported that 75%–97% of the variation in antibiotic use was located at the GP level, thus suggesting the considerable influence that differences between GPs may have on the variation. However, several study limitations render interpretation of these results delicate. First, no patient or consultation level data was used in the analysis. Although our performance data was adjusted for patient sex and age prior to this study, the results do not fully account for individual differences between patients or consultations. Previous studies, including ones conducted in Italy, have shown that patient characteristics including age, sex, type of symptoms/diagnosis, preferences, socio‐economic status and per capita income influenced antibiotic prescribing.^6^, ^11^, ^13^, ^21^, ^22^, ^36^ Second, although adjusting the model for explanatory variables did not induce a significant change in the location of the variation, it is worth noting that a limited number of variables were included in the model at the GP and AFT level, thus preventing any firm conclusion to be made as to the origin of the variation. However, our findings did indicate that the inclusion of variables induced changes to the total variance of use for several types of antibiotics. Third, this study made use of antibiotic dispensing data that did not distinguish between antibiotics prescribed by a GP and those purchased over the counter.

The creation of AFTs was intended to harmonize primary care delivery and minimize variation by fostering a collaborative environment among GPs. Researchers have suggested that group practices such as AFTs lead to practitioners influencing and depending on one another for treatment decisions.^37^ As in any other social system, norms may develop within groups of practitioners. This process of socialization leads practitioners within the same partnership to adopt similar medical behaviours and practice styles. Consequently, medical practice variation would be more due to contextual differences between group practices rather than individual differences in practice styles between practitioners. Our findings, however, suggest that little of the variation was located at the AFT level. One explanation for this is the general lack of visibility to other practitioners that prescription of medicines implies.^38^ Another explanation could be professional etiquette, where by, doctors respect the clinical autonomy of their colleagues and do not interfere with their treatment choices. The concept of professional etiquette was further illustrated by Pedersen and Jepsen's study, where Danish GPs were aware of their colleagues' prescribing behaviour but considered inappropriate to comment or criticize their colleagues' treatment choices.^39^ Our results suggest that the greatest source of variation was at the GP level, which is line with several Italian and international studies that have indicated the predominant role that GPs play regarding the variation in antibiotic prescribing in primary care.^6^, ^10^, ^11^, ^21^, ^40^, ^41^

Furthermore, our findings also suggest that the AFTs had a considerable influence over the use of certain antibiotics, namely amoxicillin‐based antibiotics. As mentioned above, amoxicillin plus clavulanic acid combinations are broad spectrum and contribute to the rise AMR, and as such, the social context in which certain AFT‐affiliated GPs find themselves in may favour the prescription of amoxicillin alone over combinations of amoxicillin with clavulanic acid.

Districts, on the other hand, seem to have a significant influence on the variation in the use of several antibiotics, namely fluoroquinolones, macrolides and third‐generation cephalosporins. One of the reasons for this could be that these molecules have been deemed by the Tuscany region as antibiotics ‘to watch’ due to their use being linked to the spread of AMR.^42^ As such, the local health authorities, which are responsible for planning and setting targets, may have promoted the adoption of directives restricting the use of these antibiotics across the districts. Consequently, the extent of the districts' influence on the variation could have been reflective of the health authorities' institutional mechanism.

For nearly 2 decades, the Tuscany region has been actively seeking to reduce unwarranted variation by implementing different strategies including primary care group practices (the AFTs), public reporting of performance data and pay for performance schemes. In fact, several studies, including ones conducted in Italy, have suggested that public reporting had a positive effect on such variation.^26^, ^43^, ^44^ In the Tuscany region, performance related to antibiotic use is reported at the district, group practice as well at the GP level. In theory, reporting of individual as well as group performance to GPs should allow them to identify potential gaps and improve prescribing behaviour individually and as a group. However, as our results seem to indicate that differences between GPs are the main source of variation, one could speculate that GPs lack visibility as to their actual performance. Thus, future research should study the effects of combining increasing performance visibility with other nudging techniques, such as providing social norm feedback, which have shown to be effective in helping GPs adopt best prescribing practices.^45^ Additionally, although GPs are already subject to a pay for performance scheme in Tuscany, the proportion of their salary that is based on performance, including pharmaceutical care, represents a fraction of their income. As such, future research should investigate the effects of further incentivizing GPs for appropriate prescribing of antibiotics.

## 5 CONCLUSION

This study sought to assess the impact of different levels of primary care governance including districts, AFTs and GPs on the variation in antibiotic use in the Tuscany region. While the variation in antibiotics' consumption was found to be in large part due to differences between GPs themselves, the influence exerted by peer groups and institutional mechanisms does have a significant impact as well.

Our analysis suggests that the health districts account for around 15% of the variation in the use of certain antibiotics, namely amoxicillin based antibiotics; injectable antibiotics; fluoroquinolones; macrolides and third‐generation cephalosporins. These findings could help better refine monitoring systems and other governance mechanisms to account for the influence that each level of healthcare governance has on antibiotic use.

Future research could investigate the ways in which a managerial approach to healthcare could translate into a culture where GPs harmonize their clinical behaviour in line with best practices, leading to not only improved patient outcomes but also large cost‐savings.

## CONFLICT OF INTEREST

The authors declare that they have no conflict of interest.

## ETHICS STATEMENT

The patients' anonymous IDs enable interlinkages between databases without disclosure of the patient's identity nor any other sensitive information, thus waiving the need for such measure.

## Supporting information

Supplementary Material 1

Supplementary Material 2

## Data Availability

Data are available from authors upon reasonable request.
