# Supplementary material for: Estimating variations in the use of antibiotics in primary care: Insights from the Tuscany region, Italy
Source: Int J Health Plann Manage. 2021 Nov 20;37(2):1049–60. doi: 10.1002/hpm.3388 (PMC9299633; doi:10.1002/hpm.3388)
Supplement: Supplementary file 1 — Supplementary Material 1 [file HPM-37-1049-s001.docx]

APPENDICES

Appendix.1 Total variance according to the model’s adjustment level

| **Indicator** |  | **Total variance** | | | | |
| --- | --- | --- | --- | --- | --- | --- |
|  |  | **Model 1 (empty model)** | | **Model 2 (with level-1 explanatory variables)** | | **Model 3 (with level-1 plus level-2 explanatory variables)** |
| **Total consumption of antibiotics expressed in DDD per 1000 inhabitants per day** | 0.507 | | 0.4210568 | | 0.4179457 | |
| **Consumption of fluoroquinolones expressed in DDD per 1000 inhabitants per day** | 0.4179457 | | 0.511269 | | 0,5104548 | |
| **Incidence of injectable antibiotics** | 1.6036208 | | 1.51710490 | | 1,49927810 | |
| **Percentage of amoxicillin based antibiotics among all amoxicillin and amoxicillin plus clavulanic acid combination based antibiotics** | 135.0669 | | 134.44447 | | 132,03475 | |
| **Percentage of fluoroquinolones among all antibiotics** | 24.1763049 | | 22.5918613 | | 22,4069477 | |
| **Percentage of macrolides among all antibiotics** | 37.824221 | | 37.794468 | | 37,65646 | |
| **Percentage of cephalosporin (3^rd^ generation antibiotics) among all antibiotics** | 16.364492 | | 16.1815716 | | 16,017991 | |

Appendix.2 Multilevel analysis: Fixed effects

|  | **Model 1 (empty model)** | | | **Model 2 (with level-1 explanatory variables)** | | | **Model 3 (with level-1 plus level-2 explanatory variables)** | | | |
| --- | --- | --- | --- | --- | --- | --- | --- | --- | --- | --- |
|  | **Coefficient** | **S.E.** | | **Coefficient** | | **S.E.** | **Coefficient** | | | **S.E.** |
| **Total consumption of antibiotics expressed in DDD per 1000 inhabitants per day** |  |  | |  | |  |  | | |  |
| **Intercept** | 2.904705 | | .0273865* | 5.344243 | .2397069 | | -.0978968 | 2.329769 | | |
| **GP level** |  | |  |  |  | |  |  | | |
| **GP age** | - | | - | -.0338932 | .0018922* | | -.0343291 | .0019398* | | |
| **Patient age** | - | | - | -.0028128 | .004142 | | .0007658 | .0043843 | | |
| **GP sex (Female)** | - | | - | .0693606 | .0297236* | | .0743857 | .0297396* | | |
| **Number of patients per GP** | - | | - | -.0002063 | .0000325* | | -.0002201 | .0000332* | | |
| **AFT level** |  | |  |  |  | |  |  | | |
| **GP age** | - | | - | - | - | | .0100351 | .0082877 | | |
| **Patient age** | - | | - | - | - | | .1322137 | .0594031* | | |
| **GP number per AFT coefficient** | - | | - | - | - | | -.0162549 | .0078995* | | |
| **% patient over 65** | - | | - | - | - | | -.081313 | .0298883* | | |
| **Number of patients per AFT** | - | | - | . | - | | .0000141 | 6.84e-06* | | |
| **Consumption of fluoroquinolones expressed in DDD per 1000 inhabitants per day** |  |  | |  | |  |  | |  | |
| **Intercept** | .9136074 | .0325412* | | 2.038805 | | .2642472* | -3.796561 | | 2.630948 | |
| **GP level** |  |  | |  | |  |  | |  | |
| **GP age** | - | - | | -.0329684 | | .0020769* | -.0336702 | | .0021311* | |
| **Patient age** | - | - | | .0203538 | | .0045542* | .0228408 | | .004812* | |
| **GP sex (Female)** | - | - | | .0262067 | | .0326028 | .0296007 | | .0326476 | |
| **Number of patients per GP** | - | - | | -.0001675 | | .0000359* | -.0001731 | | .0000367* | |
| **AFT level** |  |  | |  | |  |  | |  | |
| **GP age** | - | - | | - | | - | .0148068 | | .0092206 | |
| **Patient age** | - | - | | - | | - | .1346611 | | .0670394* | |
| **GP number per AFT coefficient** | - | - | | - | | - | -.0089898 | | .0087084 | |
| **% patient over 65** | - | - | | - | | - | -.0796048 | | .0336049* | |
| **Number of patients per AFT** | - | - | | . | | - | 7.36e-06 | | 7.51e-06 | |
| **Percentage of injectable antibiotics** |  |  | |  | |  |  | |  | |
| **Intercept** | 2.424583 | .0956977* | | -2.213083 | | .4531416* | -3.508397 | | 5.976195 | |
| **GP level** |  |  | |  | |  |  | |  | |
| **GP age** | - | - | | .0095439 | | .0034315* | .0101111 | | .0034859* | |
| **Patient age** | - | - | | .0800495 | | .0077387* | .0825493 | | .0079821* | |
| **GP sex (Female)** | - | - | | -.1087315 | | .0535378* | -.1015613 | | .0536171 | |
| **Number of patients per GP** | - | - | | -.0001393 | | .0000605* | -.0001421 | | .0000613* | |
| **AFT level** |  |  | |  | |  |  | |  | |
| **GP age** | - | - | | - | | - | -.0160498 | | .0200215 | |
| **Patient age** | - | - | | - | | - | .0813539 | | .1515574 | |
| **GP number per AFT coefficient** | - | - | | - | | - | .0028247 | | .019507 | |
| **% patient over 65** | - | - | | - | | - | -.0686995 | | .0754475 | |
| **Number of patients per AFT** | - | - | | . | | - | -.0000112 | | .0000167 | |
| **Percentage of amoxicillin based antibiotics among all amoxicillin and amoxicillin plus clavulanic acid combination based antibiotics** |  |  | |  | |  |  | |  | |
| **Intercept** | 14.29807 | .9902956* | | 24.58309 | | 4.148323* | 36.24356 | | 75.92209 | |
| **GP level** |  |  | |  | |  |  | |  | |
| **GP age** | - | - | | -.0723903 | | .0311772* | -.0654375 | | .0314248* | |
| **Patient age** | - | - | | -.1297571 | | .0706319 | -.1324156 | | .0718298 | |
| **GP sex (Female)** | - | - | | -.2632509 | | .4834909 | -.2468537 | | .4839733 | |
| **Number of patients per GP** | - | - | | .0009464 | | .0005497 | .0009333 | | .0005531 | |
| **AFT level** |  |  | |  | |  |  | |  | |
| **GP age** | - | - | | - | | - | -.5080493 | | .2537858* | |
| **Patient age** | - | - | | - | | - | .4335791 | | 1.924453 | |
| **GP number per AFT coefficient** | - | - | | - | | - | .1493686 | | .2552505 | |
| **% patient over 65** | - | - | | - | | - | -.1693957 | | .9591746 | |
| **Number of patients per AFT** | - | - | | . | | - | -.0000921 | | .0002194 | |
| **Percentage of fluoroquinolones among all antibiotics** |  |  | |  | |  |  | |  | |
| **Intercept** | 14.77526 | .3723274* | | .1464856 | | 1.725833 | -13.0989 | | 21.0291 | |
| **GP level** |  |  | |  | |  |  | |  | |
| **GP age** | - | - | | .0237999 | | .0132402 | .0217454 | | .0134897 | |
| **Patient age** | - | - | | .2533034 | | .0294266* | .2423524 | | .0305256* | |
| **GP sex (Female)** | - | - | | -.8008303 | | .2076939* | -.8154759 | | .2080592* | |
| **Number of patients per GP** | - | - | | -.0000232 | | .0002286 | .0000706 | | .0002323 | |
| **AFT level** |  |  | |  | |  |  | |  | |
| **GP age** | - | - | | - | | - | .0472832 | | .0709631 | |
| **Patient age** | - | - | | - | | - | .2447506 | | .5339109 | |
| **GP number per AFT coefficient** | - | - | | - | | - | .1140476 | | .0681365 | |
| **% patient over 65** | - | - | | - | | - | -.0690561 | | .2657882 | |
| **Number of patients per AFT** | - | - | | . | | - | -.0001014 | | .0000585 | |
| **Percentage of macrolides among all antibiotics** |  |  | |  | |  |  | |  | |
| **Intercept** | 18.22346 | .3490785* | | 21.50878 | | 2.281458* | -6.811928 | | 28.75297 | |
| **GP level** |  |  | |  | |  |  | |  | |
| **GP age** | - | - | | .0037877 | | .0176307 | .0047408 | | .0179309 | |
| **Patient age** | - | - | | -.0631605 | | .0392452 | -.0746133 | | .0407334 | |
| **GP sex (Female)** | - | - | | .1946703 | | .2762691 | .1796305 | | .2769049 | |
| **Number of patients per GP** | - | - | | -.0001969 | | .000305 | -.00021 | | .0003093 | |
| **AFT level** |  |  | |  | |  |  | |  | |
| **GP age** | - | - | | - | | - | -.0137902 | | .0987663 | |
| **Patient age** | - | - | | - | | - | .7084756 | | .7308923 | |
| **GP number per AFT coefficient** | - | - | | - | | - | -.0793253 | | .0969209 | |
| **% patient over 65** | - | - | | - | | - | -.2761652 | | .3659455 | |
| **Number of patients per AFT** | - | - | | . | | - | .0000716 | | .0000836 | |
| **Percentage of cephalosporins (3rd generation antibiotics) among all antibiotics** |  |  | |  | |  |  | |  | |
| **Intercept** | 7.969069 | .3118864* | | .9491244 | | 1.460984 | 21.34351 | | 20.10961 | |
| **GP level** |  |  | |  | |  |  | |  | |
| **GP age** | - | - | | .0140909 | | .0110643 | .0170443 | | .0112232 | |
| **Patient age** | - | - | | .1342362 | | .0248297* | .1439377 | | .0255298* | |
| **GP sex (Female)** | - | - | | -.0670568 | | .1733598 | -.0451708 | | .1735557 | |
| **Number of patients per GP** | - | - | | -.0008168 | | .0001928* | -.0008594 | | .000195* | |
| **AFT level** |  |  | |  | |  |  | |  | |
| **GP age** | - | - | | - | | - | -.1022406 | | .067185 | |
| **Patient age** | - | - | | - | | - | -.3077762 | | .5099479 | |
| **GP number per AFT coefficient** | - | - | | - | | - | -.0205189 | | .0655508 | |
| **% patient over 65** | - | - | | - | | - | .0652049 | | .2536742 | |
| **Number of patients per AFT** | - | - | | . | | - | 5.52e-06 | | .0000562 | |

* p<0.05

Appendix.3 Multilevel Model: Random effects – Variance estimates

|  | **Model 1 (empty model)** | | **Model 2 (with level-1 explanatory variables)** | | **Model 3 (with level-1 plus level-2 explanatory variables)** | | |
| --- | --- | --- | --- | --- | --- | --- | --- |
|  | **Variance**  **Component** | **S.E.** | **Variance**  **Component** | **S.E.** | **Variance**  **Component** | | **S.E.** |
| **Total consumption of antibiotics expressed in DDD per 1000 inhabitants per day** |  |  |  |  |  |  | |
| **Health district level variance** | .0133906 | .0059899* | .0125167 | .0055995* | .0126794 | .0056109* | |
| **AFT level variance** | 6.40e-07 | 2.68e-06* | .0030019 | .0030669* | .000479 | .002625* | |
| **Consumption of fluoroquinolones expressed in DDD per 1000 inhabitants per day** |  |  |  |  |  |  | |
| **Health district level variance** | .0204596 | .0074093* | .0220979 | .0079367* | .0241932 | .0088885* | |
| **AFT level variance** | 4.66e-10 | 1.71e-09* | .0026403 | .0034825* | .0002382 | .003006* | |
| **Percentage of injectable antibiotics** |  |  |  |  |  |  | |
| **Health district level variance** | .2062956 | .0646627* | .2006729 | .0630075* | .1878381 | .0594343* | |
| **AFT level variance** | .0464952 | .0163749* | .048891 | .0163179* | .044251 | .0155259* | |
| **Percentage of amoxicillin based antibiotics among all amoxicillin and amoxicillin plus clavulanic acid combination based antibiotics** |  |  |  |  |  |  | |
| **Health district level variance** | 20.37555 | 6.899782* | 20.26456 | 6.867085* | 18.22518 | 6.477724* | |
| **AFT level variance** | 13.37012 | 2.683939* | 13.27941 | 2.670496* | 12.92047 | 2.628021* | |
| **Percentage of fluoroquinolones among all antibiotics** |  |  |  |  |  |  | |
| **Health district level variance** | 3.173982 | .9867735* | 2.679247 | .8364764* | 2.56428 | .8132871* | |
| **AFT level variance** | .5231829 | .2190994* | .4569543 | .2023616* | .3868077 | .194106* | |
| **Percentage of macrolides among all antibiotics** |  |  |  |  |  |  | |
| **Health district level variance** | 2.415598 | .8757914* | 2.447919 | .8896915* | 2.345797 | .8609041* | |
| **AFT level variance** | 1.105323 | .400335* | 1.123549 | .4037372* | 1.085941* | .3994124* | |
| **Percentage of cephalosporins (3rd generation antibiotics) among all antibiotics** |  |  |  |  |  |  | |
| **Health district level variance** | 2.187502 | .6914693* | 2.238693 | .710183* | 2.131266 | .685748* | |
| **AFT level variance** | .54273 | .1774068* | .5944986 | .185498* | .5463847 | .1767404* | |

* p<0.05
